# EGFR signaling augments TLR4 cell surface expression and function in macrophages via regulation of Rab5a activation

**DOI:** 10.1007/s13238-019-00668-8

**Published:** 2019-11-08

**Authors:** Jing Tang, Bowei Zhou, Melanie J. Scott, Linsong Chen, Dengming Lai, Erica K. Fan, Yuehua Li, Qiang Wu, Timothy R. Billiar, Mark A. Wilson, Ping Wang, Jie Fan

**Affiliations:** 1 The Department of Anesthesiology, Affiliated hospital of Guangdong Medical University, Zhanjiang, 524000 China; 2 Department of Surgery, University of Pittsburgh School of Medicine, Pittsburgh, PA 15213 USA; 3 Research and Development, Veterans Affairs Pittsburgh Healthcare System, Pittsburgh, PA 15240 USA; 4 Department of Anesthesiology, Nanfang Hospital, Southern Medical University, Guangzhou, 510515 China; 5 Department of Thoracic Surgery, Shanghai Pulmonary Hospital, Tongji University School of Medicine, Shanghai, 200433 China; 6 Department of Cardiovascular Surgery, The Children’s Hospital of Zhejiang University School of Medicine, Hangzhou, 310052 China; 7 University of Pittsburgh The Graduate School of Public Health, Pittsburgh, PA 15213 USA; 8 Laboratory of Tropical Biomedicine and Biotechnology, School of Tropical Medicine and Laboratory Medicine, Hainan Medical University, Haikou, 571199 China; 9 The Feinstein Institute for Medical Research, Manhasset, NY 11030 USA; 10 McGowan Institute for Regenerative Medicine, University of Pittsburgh, Pittsburgh, PA 15219 USA; 11 Key Laboratory of Organ Injury/Protection and Translational Medicine of Zhanjiang, Zhanjiang, 524000 China

**Dear Editor**,

Toll-like receptor 4 (TLR4) is a key receptor sensing bacterial lipopolysaccharide (LPS), and is the most investigated member of the Toll-like receptor family (Kawai and Akira, 2007; Kayagaki et al., 2013; Klein et al., 2015). Cell surface TLR4 expression is determined by the balance between receptor trafficking from the Golgi apparatus to the cell membrane, and internalization of the cell surface receptor into endosomal compartments (Saltoh, 2009). In bone marrow-derived macrophages (BMDM), we observed LPS-induced EGFR phosphorylation on the surface of BMDM, and this was inhibited by pretreatment with PD168393 or TAPI-1 (Fig. S1). Next, we measured dynamic changes in cell surface TLR4 expression after LPS treatment. At 6, 12, and 24 h after LPS treatment, TLR4 expression on the surface of BMDM was increased ~2-, ~6-, and ~9-fold, respectively, as compared with controls. EGFR inhibitor PD168393, however, inhibited LPS-mediated increases in cell surface expression of TLR4 at all time points (Fig. 1A and 1B). These alterations were also confirmed in a mouse macrophage cell line, RAW264.7 cells (Fig. S2). Then, C57BL/6 mice were injected intraperitoneally (i.p.) with LPS (10 mg/kg) with or without pretreatment of EGFR inhibitor, erlotinib (100 mg/kg B.W., gavage administration) at 30 min prior to LPS. At 24 h after LPS treatment, peritoneal macrophages were collected. LPS induced 5-fold increases in TLR4 expression on the macrophage surface, and erlotinib pretreatment inhibited TLR4 cell surface expression in response to LPS (Fig. 1C and 1D). Then, BMDM cells from *EGFR*^−/−^ mice were treated with LPS for 24 h and LPS treatment failed to induce increased cell surface expression of TLR4 in *EGFR*^−/−^ BMDM compared with WT BMDM cells (Fig. 1E and 1F). Similar findings were shown *in vivo* (Fig. 1G and 1H), where in contrast to WT mice, TLR4 surface expression on peritoneal macrophages from *EGFR*^−/−^ mice was not increased at 24 h after LPS challenge. These findings indicate that EGFR phosphorylation is essential for LPS-induced up-regulation of TLR4 cell surface expression in macrophages.

**Figure 1:**
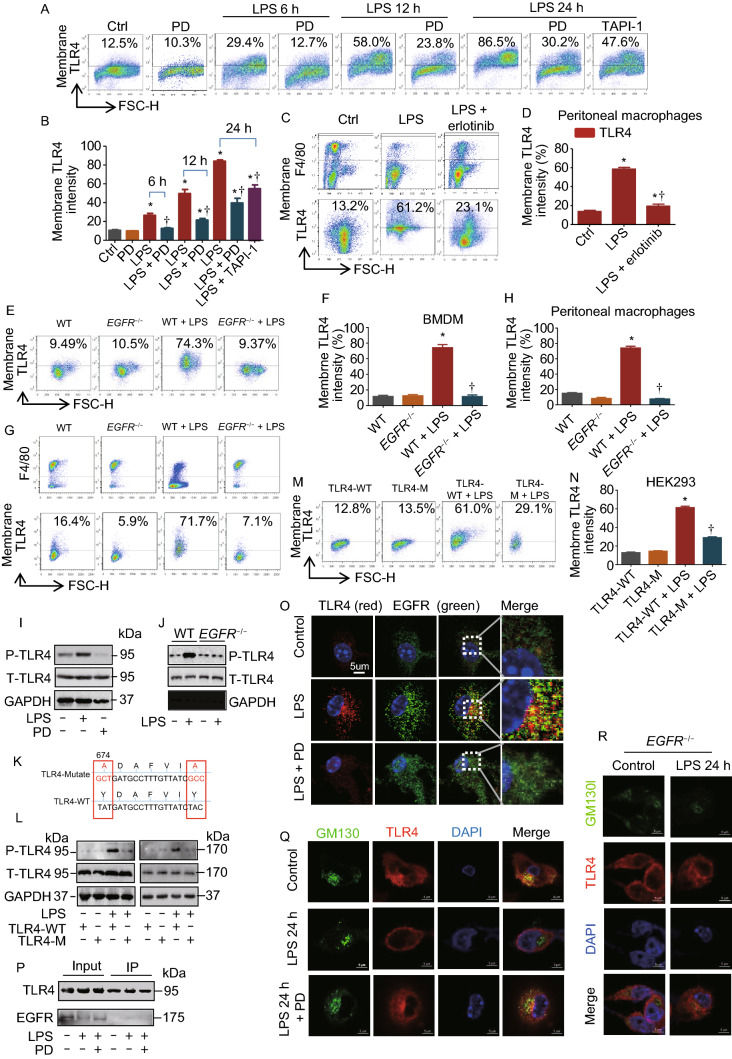
EGFR activation promotes TLR4 phosphorylation and cell surface expression of TLR4 in response to LPS. (A and B) BMDM were treated with LPS (1 μg/mL) for 6, 12, or 24 h in the presence or absence of pretreatment of PD or TAPI-1. (A) Flow cytometry analysis of cell surface TLR4 intensity in BMDM. (B) Flow cytometry analysis of cell surface TLR4 intensity in BMDM. (C and D) WT (C57BL/6) mice were treated with LPS (10 mg/kg, i.p.). In some groups, mice were pretreated with erlotinib (100 mg/kg, gavage administration) at 30 min prior to LPS i.p. Peritoneal lavage fluids were collected at 24 h after LPS treatment and peritoneal macrophages were identified with F4/80. TLR4 intensity on the surface of peritoneal macrophage was analyzed by flow cytometry. (E and F) BMDM isolated from WT and *EGFR*^−/−^ mice were treated with LPS (1 μg/mL) *in vitro* for 1 h followed by flow cytometry analysis of cell surface TLR4 intensity. (G and H) WT (C57BL/6) and *EGFR*^−/−^ mice were treated with LPS (10 mg/kg, i.p.) for 24 h. Peritoneal lavage fluids were collected, and peritoneal macrophages were identified with F4/80. TLR4 intensity on the surface of peritoneal macrophage was analyzed by flow cytometry. (I) Western blot analysis of phosphor-TLR4 in BMDM treated with LPS (1 μg/mL) for 30 min with or without PD168393 (PD, 10 μmol/L) pretreatment for 30 min. (J) Western blot analysis of phosphor-TLR4 in *EGFR*^−/−^ BMDM treated with LPS (1 μg/mL) for 30 min. (K–N) HEK293 cells were transfected with *TLR4*, *MD2*, *CD14*, *EGFR*, or *TLR4* mutant for 48 h, with treatment of LPS (1 μg/mL) for 30 min or 24 h. (K) Diagram of the TLR4 phosphorylation site mutated plasmid. (L) Western blot analysis of the phosphor-TLR4 and phosphor-EGFR in transfected HEK293 treated with LPS for 30 min. (M and N) Flow cytometry analysis of cell surface TLR4 intensity in transfected HEK293 treated with LPS for 24 h. (O and P) BMDM were treated with LPS (1 μg/mL) for 30 min with or without PD168393 (PD) pretreatment for 30 min. (O) Immune-staining of TLR4 and EGFR in BMDM. (P) Co-immunoprecipitation of TLR4 with EGFR in BMDM. (Q) Immune-staining of TLR4 and GM130 in BMDM treated with LPS (1 μg/mL) for 24 h with or without PD168393 pretreatment for 30 min. (R) Immune-staining of TLR4 and GM130 in *EGFR*^−/−^ BMDM treated with LPS (1 μg/mL) for 24 h. All images and flow cytometric plots are the representatives from at least 4 experiments. The graphs depict mean ± SD of four to six experiments or mice. *P < 0.05 as compared with control group; †*P* < 0.05 as compared with the time-matched LPS alone group

EGFR phosphorylation inhibitor, PD168393, effectively suppressed LPS-induced TLR4 phosphorylation (Fig. 1I). In addition, LPS-induced TLR4 phosphorylation was dramatically decreased in *EGFR*^−/−^ BMDM (Fig. 1J). We mutated *TLR4* 674 and 688 Tyr phosphorylation site into Ala. Then HEK293 cells were transfected with *MD2*, *CD14*, *EGFR* and *TLR4* or *TLR4* mutant. LPS treatment could not lead to the phosphorylation of EGFR in *TLR4* mutant group (Fig. 1K and 1L). We also measured the effect of TLR4 phosphorylation on TLR4 cell membrane expression. 24 h after LPS treatment LPS-induced cell surface expression of TLR4 was markedly decreased in *TLR4* mutant-expressing cells compared with cells expressing WT-TLR4 (Fig. 1M and 1N). LPS also induced co-localization of TLR4 and EGFR in BMDM at 30min after LPS treatment, and this was suppressed by EGFR phosphorylation inhibitor PD168393 (Fig. 1O). In addition, this kind of co-localization between TLR4 and EGFR in response to LPS also depended on the phosphorylation of TLR4 (Fig. S3). However, EGFR did not co-immunoprecipitate with TLR4 in control, LPS, or LPS plus PD168393 pretreatment (Fig. 1P). We further found that LPS significantly increased EGFR, but not TLR4, mRNA and total protein expression at 6, 12 and 24 h, and this was inhibited by PD168393 pretreatment (Fig. S4A–D). A large proportion of TLR4 receptors are stored in subcellular compartments, such as the Golgi apparatus and endosomes (Husebye et al., 2006). Since EGFR inhibitor decreased cell surface but not total TLR4 expression in response to LPS, we hypothesized that EGFR phosphorylation contributes to the transportation of TLR4 from the Golgi apparatus to the cell surface. Golgi marker GM130 applied to visualize the spatial relationship between the Golgi apparatus and TLR4 in BMDM. As shown in Fig. 1Q, LPS treatment reduced the co-localization of GM130 and TLR4, and PD168393 pretreatment partially restored the co-localization between Golgi and TLR4. Meanwhile, LPS lost its ability to reduce the co-localization between GM130 and TLR4 in *EGFR*^−/−^ BMDM (Fig. 1R). These data suggested that TLR4 is transported from Golgi to cell surface following LPS treatment and this is regulated by EGFR phosphorylation.

Rab5a is an important downstream signaling molecule of EGFR and plays a critical role in actin remodeling, TLR4-MyD88 interaction, and receptor internalization (Chen et al., 2012; Langemeyer et al., 2018). LPS increased Rab5a expression at both mRNA and protein levels, and PD168393 pretreatment suppressed Rab5a protein level after LPS treatment (Fig. 2A and 2B). Importantly, knockdown of *Rab5a* in BMDM significantly decreased cell surface expression of TLR4 in response to LPS (Fig. 2C and 2D). Clathrin is an important endocytic coat protein known to be involved in Rab5a mediated internalization of a variety of transmembrane receptors and their ligands (Lee et al., 2013). Pretreatment of BMDM with a clathrin inhibitor, chlorpromazine (CPZ, 12.5 μmol/L) given 30 min prior to LPS effectively inhibited the cell surface expression of TLR4 at 24 h after LPS treatment (Fig. 2E and 2F). We further determined the effect of Rab5a and clathrin on the cell surface expression of TLR4 at early time points after LPS treatment. As shown in Fig. 2G and 2H, LPS treatment for 1 h induced a significant decrease in BMDM cell surface expression. However, inhibition of EGFR or clathrin by PD168393 and CPZ, respectively, effectively suppressed the LPS-induced decrease in cell surface expression of TLR4. Knockdown of *Rab5a* also prevented decreased in TLR4 cell surface expression at 1 h after LPS treatment (Fig. 2I and 2J). In BMDM cells from *Rab5a*^−/−^ mice, as compared with WT BMDM cells, Rab5a deficiency not only effectively prevented the decrease in cell surface expression of TLR4 at 1 h after LPS treatment, but also inhibited the increase in the TLR4 cell surface expression at 24 h after LPS treatment (Fig. 2K and 2L). PD168393 pretreatment attenuated the colocalization between early endosomes and TLR4 at 1 h after LPS treatment (Fig. 2M). Furthermore, we identified co-localization between Rab5a and TLR4 near the plasma membrane of BMDM at 1 h after LPS treatment and PD168393 pretreatment prevented this colocalizations (Fig. 2N and 2O). Collectively, these results indicate that EGFR-dependent initiation of internalization of TLR4, which is mediated by clathrin and Rab5a, consequently enhanced late phase cell surface expression of TLR4 in response to LPS.

**Figure 2:**
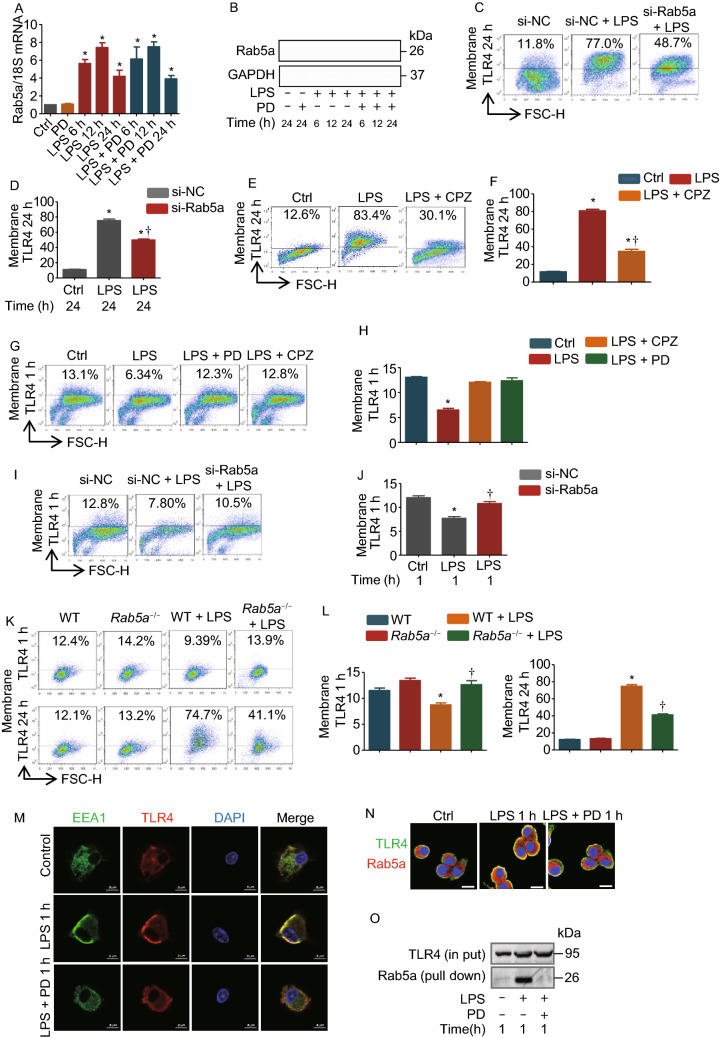
Rab5a-mediated concurrent internalization of TLR4 and EGFR results increased cell surface expression of the receptors. (A and B) BMDM were treated with LPS for 6, 12, or 24 h in the presence or absence of pretreatment of PD168393 (PD) for 30 min. (A) Real time PCR analysis of Rab5a expression. (B) Western blot analysis of Rab5a expression. (C and D) BMDM transfected with si-NC and si-Rab5a for 48 h were treated with LPS (1 μg/mL) for 24 h. Flow cytometry analysis of cell surface TLR4. (E–H) BMDM were treated with LPS (1 µg/mL) for 1 h or 24 h, with or without clathrin inhibitor chlorpromazine (CPZ 12.5 μmol/L) or PD168393 (PD 10 μmol/L) pretreatment for 30 min. Flow cytometry analysis of cell surface TLR4 at 24 h or after LPS. (I and J) BMDM cells transfected with si-NC or si-Rab5a for 48 h followed by LPS treatment (1 μg/mL) for 1 h. Flow cytometry analysis of cell surface TLR4. (K and L) WT and *Rab5a*^−/−^ BMDM were treated with LPS (1 μg/mL) for 1 h or 24 h. Flow cytometry analysis of cell surface TLR4 at 1 h or 24 h after LPS. (M–O) BMDM were treated with LPS (1 μg/mL) for 1 h with or without PD168393 (PD) pretreatment for 30 min. (M) Immune-staining of TLR4 and EEA1 in BMDM. (N) Immune-staining of TLR4 with Rab5a. (O) Co-immunoprecipitation between TLR4 and Rab5a in BMDM. All flow cytometric plots are the representative from at least 4 experiments. The graphs depict mean ± SD of four to six experiments or mice. *P < 0.05 as compared with control group; †*P* < 0.05 as compared with the time-matched LPS alone group

Epidermal growth factor receptor pathway substrate 8 (EPS8)/related to the N-terminus of tre oncogene (RN-TRE) and growth-factor receptor-bound protein 2 (GRB2)/Ras and Rab interactor 1 (RIN1) have been reported to coordinate the function of Rab5 GEFs and GTPase-activating proteins (GAPs) for the maintenance of normal trafficking of cell membrane receptors (Mendelsohn and Baselga, 2006; Chen et al., 2012). LPS increased EPS8 and GRB2 mRNA and protein expression, and this was significantly inhibited by PD168393 pretreatment (Fig. S5A, S5B and S5E). LPS also increased RN-TRE expression, but this was not suppressed by PD168393 (Fig. S5C and S5D). LPS did not affect the expression of RIN1 (Fig. S5C and S5E). We further demonstrated using coimmunoprecipitation that GRB2 and EPS8 associated with TLR4 at 6 h after LPS treatment (Fig. S5F), and these findings were visualized by immunofluorescence imaging in BMDM cells (Fig. S5G). We found knockdown of any of *EPS8*/*RN-TRE*/*GRB2*/*RIN1*significantly suppressed cell surface expression of TLR4 at 24 h following LPS treatment (Fig. S5H and S5I), suggesting that EPS8/RN-TRE/GRB2/RIN1 serve as a signaling pathway mediating activation of Rab5a and subsequent upregulation of macrophage surface expression of TLR4 and EGFR in response to LPS.

We measured LPS binding to the cell, detected activation of p38, and ERK1/2 as the downstream signaling of TLR4, and measured cytokine release from the macrophages. Binding of Alexa Fluor®488-conjugated LPS to BMDM was gradually increased over the 24 h after LPS treatment, and PD168393 significantly decreased LPS binding (Fig. S6A and S6B). LPS significantly promoted the phosphorylation of p38 and ERK1/2 at 6, 12 and 24 h after LPS treatment, and these changes in phosphorylation were partially suppressed by PD168393 pretreatment (Fig. S6C). PD168393 also inhibited LPS-induced reactive oxygen species (ROS) production in BMDM at 12 h and 24 h after LPS (Fig. S6D–E). Lastly, we demonstrated that PD168393 markedly inhibited LPS-induced expression of *IL-1β*, *IL-10*, *IL-6* and *TNF-α* in both BMDM and RAW264.7 cells at 24 h after LPS treatment (Figs. S6F and S7). Furthermore, knockdown of *EPS8*, *GRB2*, or *Rab5a* in BMDM suppressed LPS-induced cytokine expression at 24 h (Fig. 6G) and phosphorylation of p38 and ERK1/2 (Fig. S6H). In addition, LPS failed to induce phosphorylation of p38 and ERK1/2 in BMDM isolated from *EGFR*^−/−^ or *Rab5a*^−/−^ mice (Fig. S6I), and failed to induce the expression of cytokines (Fig. S6J).

RAW264.7 cells and BMDM were treated with LPS for 24 h followed by assessment of cell death. As shown in Fig. S8A–D, LPS increased cell death in RAW264.7 cells and BMDM and this was attenuated with PD168393 pretreatment, which prevents the up-regulation of TLR4 cell surface expression. At 24 h after LPS treatment, macrophages were collected from peritoneal lavage fluid from the mice and macrophages identified. As shown in Fig. S8E and S8F, macrophage death increased from 2.2% to 14.1% in response to LPS, and erlotinib pretreatment significantly prevented LPS-induced macrophage death. Necroptosis and pyroptosis are two major types of cell death known to be induced by LPS (Pilla et al., 2014; Li et al., 2016). At 12 h and 24 h after LPS stimulation DNA fragmentation and caspase-1 activation, as detected by flow cytometry, occurred in BMDM (Fig. S9A and S9C). Morphologically, we observed nuclear condensation and enlarged cell size plus caspase-1 activation (Fig. S9B) by using confocal microscopy at 24 h after LPS treatment. These cellular alterations are known characteristics of pyroptosis (Miao et al., 2011). EGFR phosphorylation inhibitor PD168393 suppressed this macrophage pyroptosis (Fig. S9A–D). In addition, immunoblotting and confocal microscopy showed that LPS induced association of receptor-interacting serine/threonine-protein kinase 1 (RIPK1) and RIPK3 in BMDM, a key molecular event that drives cell necroptosis (Fig. S9E and S9F).

This study elucidates a novel mechanism, in which Rab5a plays an important role in promoting macrophage surface expression of TLR4 after LPS stimulation. EGFR phosphorylation leads to the activation of its substrates EPS8 and GRB2, which, in turn, activate ras effector RIN1 and GAP protein RN-TRE. RIN1 and RN-TRE work together to coordinate the equilibrium of GTP and GDP-bound forms of Rab5a to secure the process of receptor internalization. Importantly, receptor internalization is an essential step to promoting increased cell surface expression of TLR4, as well as enhanced inflammation in response to LPS. This study, at least in part, elucidates why EGFR inhibitor is able to attenuate inflammatory responses to LPS and protect endotoxemic animals from death. In addition, during experiments, we included both male and female mice, and thus, the EGFR-mediated enrichment of TLR4 cell surface expression is not sex-dependent.

## Electronic supplementary material

Below is the link to the electronic supplementary material.

Supplementary material 1 (PDF 2419 kb)
