# Correction to: EGFR signaling augments TLR4 cell surface expression and function in macrophages via regulation of Rab5a activation

**DOI:** 10.1007/s13238-019-00679-5

**Published:** 2020-03-03

**Authors:** Jing Tang, Bowei Zhou, Melanie J. Scott, Linsong Chen, Dengming Lai, Erica K. Fan, Yuehua Li, Qiang Wu, Timothy R. Billiar, Mark A. Wilson, Ping Wang, Jie Fan

**Affiliations:** 1 The Department of Anesthesiology, Affiliated hospital of Guangdong Medical University, Zhanjiang, 524000 China; 2 Department of Surgery, University of Pittsburgh School of Medicine, Pittsburgh, PA 15213 USA; 3 Research and Development, Veterans Affairs Pittsburgh Healthcare System, Pittsburgh, PA 15240 USA; 4 Department of Anesthesiology, Nanfang Hospital, Southern Medical University, Guangzhou, 510515 China; 5 Department of Thoracic Surgery, Shanghai Pulmonary Hospital, Tongji University School of Medicine, Shanghai, 200433 China; 6 Department of Cardiovascular Surgery, The Children’s Hospital of Zhejiang University School of Medicine, Hangzhou, 310052 China; 7 University of Pittsburgh The Graduate School of Public Health, Pittsburgh, PA 15213 USA; 8 Laboratory of Tropical Biomedicine and Biotechnology, School of Tropical Medicine and Laboratory Medicine, Hainan Medical University, Haikou, 571199 China; 9 The Feinstein Institute for Medical Research, Manhasset, NY 11030 USA; 10 McGowan Institute for Regenerative Medicine, University of Pittsburgh, Pittsburgh, PA 15219 USA; 11 Key Laboratory of Organ Injury/Protection and Translational Medicine of Zhanjiang, Zhanjiang, 524000 China

## Correction to: Protein Cell 10.1007/s13238-019-00668-8

In the original publication the bands in Fig. 1J and Fig. 2B were not visible. The correct versions of Fig. 1J and Fig. 2B are provided in this correction.

**Figure 1:**
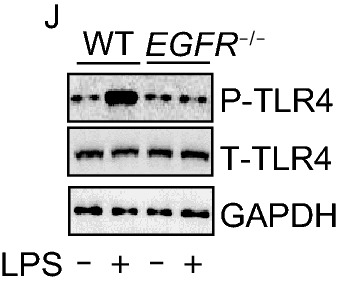
EGFR activation promotes TLR4 phosphorylation and cell surface expression of TLR4 in response to LPS. (A and B) BMDM were treated with LPS (1 μg/mL) for 6, 12, or 24 h in the presence or absence of pretreatment of PD or TAPI-1. (A) Flow cytometry analysis of cell surface TLR4 intensity in BMDM. (B) Flow cytometry analysis of cell surface TLR4 intensity in BMDM. (C and D) WT (C57BL/6) mice were treated with LPS (10 mg/kg, i.p.). In some groups, mice were pretreated with erlotinib (100 mg/kg, gavage administration) at 30 min prior to LPS i.p. Peritoneal lavage fluids were collected at 24 h after LPS treatment and peritoneal macrophages were identified with F4/80. TLR4 intensity on the surface of peritoneal macrophage was analyzed by flow cytometry. (E and F) BMDM isolated from WT and *EGFR*^−/−^ mice were treated with LPS (1 μg/mL) *in vitro* for 1 h followed by flow cytometry analysis of cell surface TLR4 intensity. (G and H) WT (C57BL/6) and *EGFR*^−/−^ mice were treated with LPS (10 mg/kg, i.p.) for 24 h. Peritoneal lavage fluids were collected, and peritoneal macrophages were identified with F4/80. TLR4 intensity on the surface of peritoneal macrophage was analyzed by flow cytometry. (I) Western blot analysis of phosphor-TLR4 in BMDM treated with LPS (1 μg/mL) for 30 min with or without PD168393 (PD, 10 μmol/L) pretreatment for 30 min. (J) Western blot analysis of phosphor-TLR4 in *EGFR*^−/−^ BMDM treated with LPS (1 μg/mL) for 30 min. (K–N) HEK293 cells were transfected with *TLR4*, *MD2*, *CD14*, *EGFR*, or *TLR4* mutant for 48 h, with treatment of LPS (1 μg/mL) for 30 min or 24 h. (K) Diagram of the TLR4 phosphorylation site mutated plasmid. (L) Western blot analysis of the phosphor-TLR4 and phosphor-EGFR in transfected HEK293 treated with LPS for 30 min. (M and N) Flow cytometry analysis of cell surface TLR4 intensity in transfected HEK293 treated with LPS for 24 h. (O and P) BMDM were treated with LPS (1 μg/mL) for 30 min with or without PD168393 (PD) pretreatment for 30 min. (O) Immune-staining of TLR4 and EGFR in BMDM. (P) Co-immunoprecipitation of TLR4 with EGFR in BMDM. (Q) Immune-staining of TLR4 and GM130 in BMDM treated with LPS (1 μg/mL) for 24 h with or without PD168393 pretreatment for 30 min. (R) Immune-staining of TLR4 and GM130 in *EGFR*^−/−^ BMDM treated with LPS (1 μg/mL) for 24 h. All images and flow cytometric plots are the representatives from at least 4 experiments. The graphs depict mean ± SD of four to six experiments or mice. *P < 0.05 as compared with control group; †*P* < 0.05 as compared with the time-matched LPS alone group

**Figure 2:**
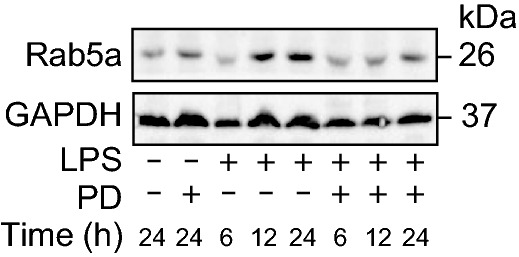
Rab5a-mediated concurrent internalization of TLR4 and EGFR results increased cell surface expression of the receptors. (A and B) BMDM were treated with LPS for 6, 12, or 24 h in the presence or absence of pretreatment of PD168393 (PD) for 30 min. (A) Real time PCR analysis of Rab5a expression. (B) Western blot analysis of Rab5a expression. (C and D) BMDM transfected with si-NC and si-Rab5a for 48 h were treated with LPS (1 μg/mL) for 24 h. Flow cytometry analysis of cell surface TLR4. (E–H) BMDM were treated with LPS (1 µg/mL) for 1 h or 24 h, with or without clathrin inhibitor chlorpromazine (CPZ 12.5 μmol/L) or PD168393 (PD 10 μmol/L) pretreatment for 30 min. Flow cytometry analysis of cell surface TLR4 at 24 h or after LPS. (I and J) BMDM cells transfected with si-NC or si-Rab5a for 48 h followed by LPS treatment (1 μg/mL) for 1 h. Flow cytometry analysis of cell surface TLR4. (K and L) WT and *Rab5a*^−/−^ BMDM were treated with LPS (1 μg/mL) for 1 h or 24 h. Flow cytometry analysis of cell surface TLR4 at 1 h or 24 h after LPS. (M–O) BMDM were treated with LPS (1 μg/mL) for 1 h with or without PD168393 (PD) pretreatment for 30 min. (M) Immune-staining of TLR4 and EEA1 in BMDM. (N) Immune-staining of TLR4 with Rab5a. (O) Co-immunoprecipitation between TLR4 and Rab5a in BMDM. All flow cytometric plots are the representative from at least 4 experiments. The graphs depict mean ± SD of four to six experiments or mice. *P < 0.05 as compared with control group; †*P* < 0.05 as compared with the time-matched LPS alone group

