# Supplementary material for: EGFR signaling augments TLR4 cell surface expression and function in macrophages via regulation of Rab5a activation
Source: Protein Cell. 2019 Nov 8;11(2):144–9. doi: 10.1007/s13238-019-00668-8 (PMC6954894; doi:10.1007/s13238-019-00668-8)
Supplement: Supplementary file 1 — Supplementary material 1 (PDF 2419 kb) [file 13238_2019_668_MOESM1_ESM.pdf]

Materials and Methods

Animals

C57BL/6 (WT) mice were purchased from Jackson Laboratory (Bar Harbor, ME, USA), *Rab5a* and *EGFR* knockout mice were purchased from Cyagen Biosciences (Guangzhou, China). All animal experimental protocols were reviewed and approved by the Institutional Animal Care and Use Committees of University of Pittsburgh and VA Pittsburgh Healthcare System, and Institutional Animal Care and Use Committees of Guangdong Medical University. Eight-week- old C57BL/6 mice with a mean body weight of 25g were used. For animal studies, the mice were divided into the following three groups: (1) control group: mice received intraperitoneal (i.p.) injections of saline and pretreatment of equal amounts of erlotinib solvent (Captisol) orally 30min before saline i.p.; (2) LPS group: mice were pretreated with equal amounts of Captisol orally 30min before LPS (10mg/kg, i.p.) treatment; (3) LPS + erlotinib group: mice were pretreated with 100mg/kg erlotinib (dissolved in Captisol) orally 30min before LPS (10mg/kg, i.p.) treatment.

Flow cytometry analysis

Macrophages collected from peritoneal lavage were labeled with F4/80 at 4℃ for 15min. For measuring cell surface expression of TLR4, macrophages were stained with PE conjugated anti-mouse CD284 (TLR4) antibody (eBioscience affymetrix, San Diego, CA, USA) for 30min. For detecting cell death, cells were incubated with 7-AAD and Annexin V (559763, BD PharmingenTM, San Jose, CA, USA) for 15min at room temperature, and the double-stained cells were counted as dead cells. For determining macrophage pyroptosis, cells were incubated with Alexa Fluor 488-conjugated caspase-1 FLICA at 37 ℃ for 1h. After being fixed with 4% paraformaldehyde, cells were then stained with TMR red-labeled In-Situ Cell Death Detection reagent (12156792910, Roche Applied Science, Indianapolis, IN, USA). Cells were analyzed by flow cytometry, and acquisition was performed on 30,000 events using a FACScalibur cytometer

(BD Biosciences, San Jose, CA, USA) and FlowJo-V10 software (Tree Star, Ashland, OR, USA). DMSO was used as the vehicle controls for PD168393 solvent.

Bone marrow derived macrophage (BMDM) isolation and culture

Bone marrow was flushed out with pre-chilled Dulbecco's Modified Eagle Medium (DMEM) from femurs and tibias, which were harvested from C57BL/6 WT mice following the methods as described previously (Chen et al., 2018).

RNA extraction and quantitative real-time PCR

Total RNA was isolated from BMDM or RAW264.7 cells using TRIzol RNA Isolation Reagents (15596026, Life Technologies, Pittsburgh, PA, USA). Real-time RT-PCR was done using the iTaq™ Universal SYBR® Green Supermix (172-5121, Bio-Rad, Hercules, CA, USA) in a Bio-Rad iQ5 real-time PCR machine (Bio-Rad). Reverse transcription was done using iScript Reverse Transcription Supermix (170-8841, Bio-Rad, Hercules, CA, USA). Amplification was performed with cycling conditions of 95 ℃ for 15s then 60 ℃ for 30s for 40 cycles. After the amplification protocol was over, PCR product was subjected to melt-curve analysis using Bio- Rad iQ5 software (Bio-Rad). The gene-specific primers were listed as below: Rab5a Forward Primer: GCTAATCGAGGAGCAACAAGAC; Reverse Primer: CCAGGCTTGATTTGCCAACAG. RN-TRE Forward Primer: GATTCCGACCAGGATGTAGCA; Reverse Primer: CTTCCCAGGGCTCGATCTCT. EPS8 Forward Primer: TCTTCACCACCCTATTCCCAG; Reverse Primer:CATCTTTCCGATCCAGCACGA. GRB2 Forward Primer: CCCTGTCCGTCAAGTTTGGAA;

Reverse Primer: GGCATCTGTTCTATGTCCCGTAA. RIIN1 Forward Primer: TATGACACACCTGATACCAGAGG; Reverse Primer: AGTGTTAGATTTCCGCACCAG.

EGFR Forward Primer: GCCATCTGGGCCAAAGATACC; Reverse Primer:

GTCTTCGCATGAATAGGCCAAT. TLR4 Forward Primer: ATGGCATGGCTTACACCACC; Reverse Primer: GAGGCCAATTTTGTCTCCACA. TNF-α Forward Primer: CCCTCACACTCAGATCATCTTCT; Reverse Primer: GCTACGACGTGGGCTACAG. IL-1β Forward Primer: GCAACTGTTCCTGAACTCAACT; Reverse Primer: ATCTTTTGGGGTCCGTCAACT. IL-10 Forward Primer: GCTCTTACTGACTGGCATGAG; Reverse Primer: CGCAGCTCTAGGAGCATGTG. IL-6 Forward Primer: TAGTCCTTCCTACCCCAATTTCC; Reverse Primer: TTGGTCCTTAGCCACTCCTTC. 18s Forward Primer: GTAACCCGTTGAACCCCATT; Reverse Primer: CCATCCAATCGGTAGTAGCG.

Gene knockdown

BMDM cells (4×10^5 cells) were cultured in 35mm dishes for 24h before transfection. 200pmol per dish of mouse EPS8 siRNA, GRB2 siRNA, RIN1 siRNA, RN-TRE siRNA, Rab5a siRNA or non-specific siRNA (Integrated DNA Technologies) was transfected using Lipofectamine LTX Plus Transfection Reagent (15338-030 Invitrogen Life Technologies) following the manufacturer’s instructions. At 48h after transfection, the efficiency of the corresponding gene knockdown was confirmed with Western blot.

Plasmid transfection

Wild type human *TLR4* (GV141-hTLR4) and mutant *TLR4* (Y674A and Y680A) (GV141-hTLR4-Y674/680A) plasmid were purchased from Genechem (Shanghai, China). HEK293 cells were transfected with control GV141, GV141-mTLR4, or GV141-mTLR4- Y674/680A plasmids (2.5μg/10^5 cells) using Lipofectamine 3000 reagent (Life Technologies, USA) according to the manufacturer’s protocol. At 24h after transfection, cells were stimulated with LPS (1ug/ml) for 24h

and then harvested for membrane TLR4 detection using flow cytometry.

Immunoprecipitation and immunoblot

Cells were suspended in IP lysis buffer (Thermo, CA, USA) at 4 ℃ for 30min with continuous mixing. After centrifugation at 12,000 × g for 15min, a final volume of 500μl supernatants (500μg protein) was precleared for 1h with 20ul of a 50% slurry of Protein A/G magnetic bead (Merck Millipore, CA, USA) at 4 ℃. We used the magnetic stand to capture the beads and incubated the supernatants with 20μl of antibodies against mouse TLR4 (Santa Cruz Biotechnologies) at 4°C overnight. 50μl of Protein A/G magnetic beads were added to the solution and incubated for an additional hour. Thereafter, the beads were collected and washed 3 times with 1ml PBS, proteins were subjected to SDS-PAGE (6–20% gel) and then transferred to Immobilon-P membranes for Western blotting.

Immunofluorescence

BMDM cells were plated in 35mm Glass Bottom Dish (801002-1, NEST, Wuxi, China) and then fixed in 4% paraformaldehyde, and permeabilized with 0.5% TritonX-100, followed by blocked with 1% BSA for 30min at room temperature. Next, the primary antibody was added and incubated with cells at 4 ℃ overnight. After washing the cells three times with PBS appropriate fluorescent secondary antibodies were added and incubated with cells for 1h at RT in the dark. Cells were washed three times again, counterstained with DAPI and then observed under a fluorescence microscope.

Antibodies and Reagents

Antibodies: RIPK1 antibody (610458, BD Biosciences, San Jose, CA, USA), RIPK3 antibody (sc-135170, Santa Cruz Biotechnology, Dallas, TX, USA), TLR4 antibody (sc-30002, Santa Cruz Biotechnology), EGFR antibody (sc-120, Santa Cruz Biotechnology, Dallas, TX, USA),

GRB2 antibody (sc-8034, Santa Cruz Biotechnology, Dallas, TX, USA), RIN1 antibody (sc-1971, Santa Cruz Biotechnology, Dallas, TX, USA), EPS8 antibody (sc-390257, Santa Cruz Biotechnology, Dallas, TX, USA), Phospho-p38 MAPK (Thr180/Tyr182) (3D7) XP Rabbit mAb (9215, Cell Signaling Technology, Danvers, MA, USA), p38 MAPK(D13E1) XP Rabbit mAb (8690, Cell Signaling Technology, Danvers, MA, USA), p44/42 MAPK (Erk1/2) (137F5) Rabbit mAb (695, Cell Signaling Technology, Danvers, MA, USA), GAPDH (D16H11) XP Rabbit mAb (5174 Cell Signaling Technology, Danvers, MA, USA), IκB-α antibody (sc-371, Santa Cruz Biotechnology, Dallas, TX, USA), Rab5a antibody (NB120-13253, Novus, Grand Island, NY, USA), RN-TRE antibody (NBP2-20839, Novus, Grand Island, NY, USA), Phospho-EGF Receptor (Tyr1068) Antibody (2234S, Cell Signaling Technology, Danvers, MA, USA),

Caspase-1 antibody (ab1872, Abcam, Cambridge, MA , USA), EEA1 Antibody(ab2900, Abcam, Cambridge, MA , USA), GM130 antibody(11308-1-AP, Proteintech, Wuhan, Hubei, China)

Reagents: Pierce Co-Immunoprecipitation Kit (26149, Thermo Fisher Scientific, Pittsburgh, PA, USA), Lipopolysaccharides from Salmonella Minnesota, Alexa Fluor® 488 conjugate (L23356, Thermo Fisher Scientific, Pittsburgh, PA, USA), Chlorpromazine hydrochloride (C-8138, Sigma, Louis, MO, USA), TACE inhibitor TAPI-1 (171235-71-5, Cayman, Ann Arbor, MI, USA), Captisol was kindly provided by ligand Technology (San Diego, CA, USA), Erlotinib hydrochloride (CDS022564, Sigma, Louis, MO, USA), EGFR phosphorylation inhibitor PD168393 (PZ0285-5MG, Sigma, Louis, MO, USA).

Statistical analysis

Results are presented as mean ± SD. Differences between two groups were analyzed by Student t test. For multi-group comparisons, One-way ANOVA was performed followed by Tukey's post-hoc test. $P<0.05$ was considered statistically significant. Graphs and figures were made with Graphpad Prism 6 (GraphPad software, CA, USA).

SUPPLEMENTARY FIGURES

Figure S1. LPS induced EGFR phosphorylation in BMDM cells. (A and B) BMDM were treated with LPS (1μg/ml) for 30 min, with or without pretreated with PD168393 (PD, 10μM) or TAPI-1 (90μM) for 30 min. (A) Western blot analysis of EGFR expression and phosphorylation in the BMDM. (B) Immunofluorescence staining of phosphor-EGFR and total-EGFR in BMDM. The graphs depict mean ± SD of three experiments. * $p < 0.05$ as compared with control group; † $p < 0.05$ as compared with the time-matched LPS alone group.

Figure S2: LPS promotes the expression of TLR4 on the cell surface of Raw264.7 cells. Raw264.7 cells were treated with LPS for 24 h in the presence or absence of pretreatment of PD168393 (PD) or TAPI-1 for 30 min. (A and B) Flow cytometry analysis of cell surface TLR4 intensity in Raw264.7. All flow cytometric plots are the representative from at least 4 experiments. The graphs depict mean ± SD of four to six experiments or mice. * $p < 0.05$ as compared with control group; † $p < 0.05$ as compared with the time-matched LPS alone group.

Figure S3: The effect of TLR4 phosphorylation on the colocalization between TLR4 and EGFR in response to LPS.

Immune-staining of TLR4 and EGFR or TLR4 mutant and EGFR in HEK293 cells transfected with *TLR4*, *MD2*, *CD14*, *EGFR*, or *TLR4* mutant for 48 h, with treatment of LPS (1μg/ml) for 30 min.

Figure S4: The effect of LPS on the expression of TLR4 and EGFR in BMDM cells. (A-D) BMDM were treated with LPS for 6, 12 and 24 h with or without PD168393 (PD) pretreatment for

30 min. (A and B) Real time PCR analysis for *TLR4* or *EGFR* mRNA expression in BMDM. (C and D) Western blot for TLR4 or EGFR protein expression in BMDM. All images are the representatives from at least 4 experiments. The graphs depict mean ± SD of four to six experiments or mice. * $p < 0.05$ as compared with control group; † $p < 0.05$ as compared with the time-matched LPS alone group.

Figure S5. EPS8/GRB2/RN-TRE/RIN1 are required for the upregulation of cell surface expression of TLR4 and EGFR in response to LPS. (A-G) BMDM were treated with LPS for 6, 12, or 24 h in the presence or absence of pretreatment of PD168393 (PD). (A-D) Real time PCR analysis of *EPS8, RN-TRE, GRB2* and *RIN1* mRNA expression in BMDM. (E) Western blot analysis of EPS8, RN-TRE, GRB2 and RIN1 protein expression in BMDM. .(F) Co-immunoprecipitation of TLR4 with EPS8/GRB2/RN-TRE/RIN1 in BMDM at 6 h after LPS treatment. (G) Immunofluorescence staining of TLR4 and EPS8 or GRB2 or RN-TRE orRIN1 in BMDM at 6 h after LPS treatment. (H and I) BMDM were treated with LPS for 24 h. In some groups, BMDM were treated with PD168393 (PD) for 30 min or pre-transfected with si-NC, si-EPS8, si-RN-TRE, si-GRB2 and si-RIN1 for 48 h before LPS treatment. Flow cytometry analysis of cell surface TLR4 intensity in BMDM. All images and flow cytometric plots are the representative from at least 4 experiments. The graphs depict mean ± SD of four to six experiments or mice. * $p < 0.05$ as compared with control group; † $p < 0.05$ as compared with the time-matched LPS alone group.

Figure S6. Upregulated cell surface TLR4 expression sensitizes cell response to LPS. (A-F) BMDM were treated with LPS for 6, 12, or 24 h in the presence or absence of pretreatment of PD168393 (PD) (A and B) Flow cytometry analysis of cell surface and internalized LPS intensity

in BMDM. (C) Western blot analysis of the expression and phosphorylation of p38 and ERK1/2 in BMDM. (D and E) Flow cytometry analysis of ROS production in BMDM. (F) Real time PCR analysis of *il-1β*, *il-10*, *il-6* and *TNF-α* mRNA expression in BMDM. (G and H) Effect of *GRB2* or *EPS8* or *Rab5a* knocking down on LPS-induced cytokine expression and P38, ERK1/2 activation in BMDM. BMDM were transfected with si-NC, si-EPS8, si-RN-TRE and si-Rab5a for 48h following by treatment of LPS (1μg/ml) for 6 h. (G) Real time PCR analysis of *il-1β*, *il-10*, *il-6* and *TNF-α* mRNA expression in BMDM. (H) Western blot analysis the expression and phosphorylation of p38 and ERK1/2 in BMDM. (I and J) BMDM isolated from WT, *Rab5a-/-* and *EGFR-/-* mice were treated with LPS (1μg/ml) in vitro for 6 h. (I) Western blot analysis the expression and phosphorylation of p38 and ERK1/2 in BMDM. (J) Real time PCR analysis of *il-1β*, *il-10*, *il-6* and *TNF-α* mRNA expression in BMDM. All Western blot photos and flow cytometric plots are the representative from at least 4 experiments. The graphs depict mean ± SD of four to six experiments or mice. * $p < 0.05$ as compared with control group; † $p < 0.05$ as compared with the time-matched LPS alone group.

Figure S7: Increased TLR4 on the cell surface is responsible for the regulatory effect of EGFR on LPS induced cytokines production in Raw 264.7 cells. (A-D) Raw264.7 cell were treated with LPS for 6, 12 and 24 h in the presence or absence of pretreatment of PD168393 (PD) for 30 min following by Real time PCR analysis of *IL-1β*, *IL-10*, *IL-6* and *TNF-α* mRNA expression. The graphs depict mean ± SD of four to six experiments or mice. * $p < 0.05$ as compared with control group; † $p < 0.05$ as compared with the time-matched LPS alone group.

Figure S8. Upregulated TLR4 expression promotes macrophage death in response to LPS. (A and B) R264.7 cells were treated with LPS (1μg/ml) for 24 h in the presence or absence of

pretreatment of PD168393 (PD) for 30 min followed by flow cytometry analysis of cell death. (C and D) BMDM cell were treated with LPS (1μg/ml) for 24 h in the presence or absence of pretreatment of PD168393 (PD) for 30 min followed by flow cytometry analysis of cell death. (E and F) WT (C57BL/6) mice were treated with LPS (10mg/kg, i.p.). In some groups, mice were pretreated with erlotinib (100mg/kg, gavage administration) at 20 min prior to LPS i.p. Peritoneal lavage fluids were collected at 24 h after LPS treatment and peritoneal macrophages were identified with F4/80. Cell death was analysis by flow cytometry. All flow cytometric plots are the representative from at least 4 experiments. The graphs depict mean ± SD of four to six experiments or mice. * $p < 0.05$ as compared with control group; † $p < 0.05$ as compared with the time-matched LPS alone group.

Figure S9. Upregulated TLR4 expression promotes macrophage necrosis in macrophages in response to LPS. (A-F) BMDM cell were treated with LPS for 12 and 24 h in the presence or absence of pretreatment of PD168393 (PD) for 30 min. (A) Western blot analysis the activation of Caspase-1 in BMDM. (B) immunofluorescence staining showing the activated Caspase-1 and TUNEL in BMDM. (C and D) Flow cytometry analysis of cell pyroptosis in BMDM. (E) Co-immunoprecipitation between RIP1 and RIP3 in BMDM. (F) immunofluorescence staining of RIP1 and RIP3 in BMDM. All images and flow cytometric plots are the representative from at least 4 experiments. The graphs depict mean ± SD of four to six experiments or mice. * $p < 0.05$ as compared with control group; † $p < 0.05$ as compared with the time-matched LPS alone group.

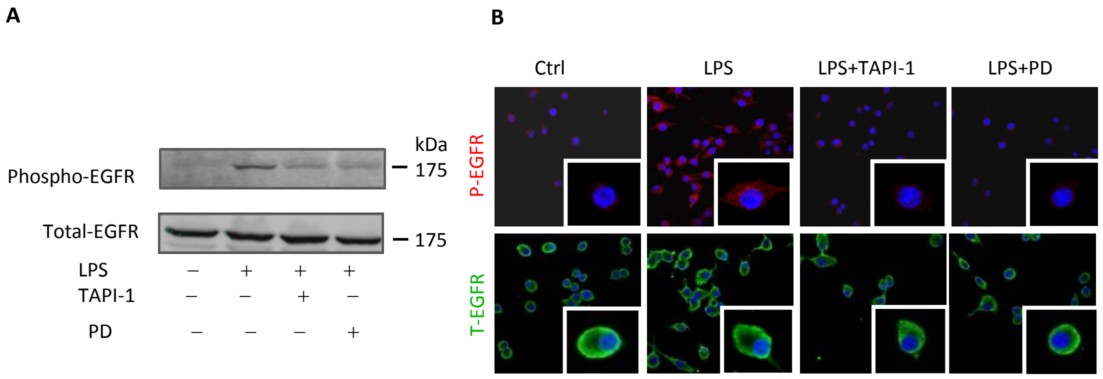

Figure S1. LPS induced EGFR phosphorylation in BMDM cells

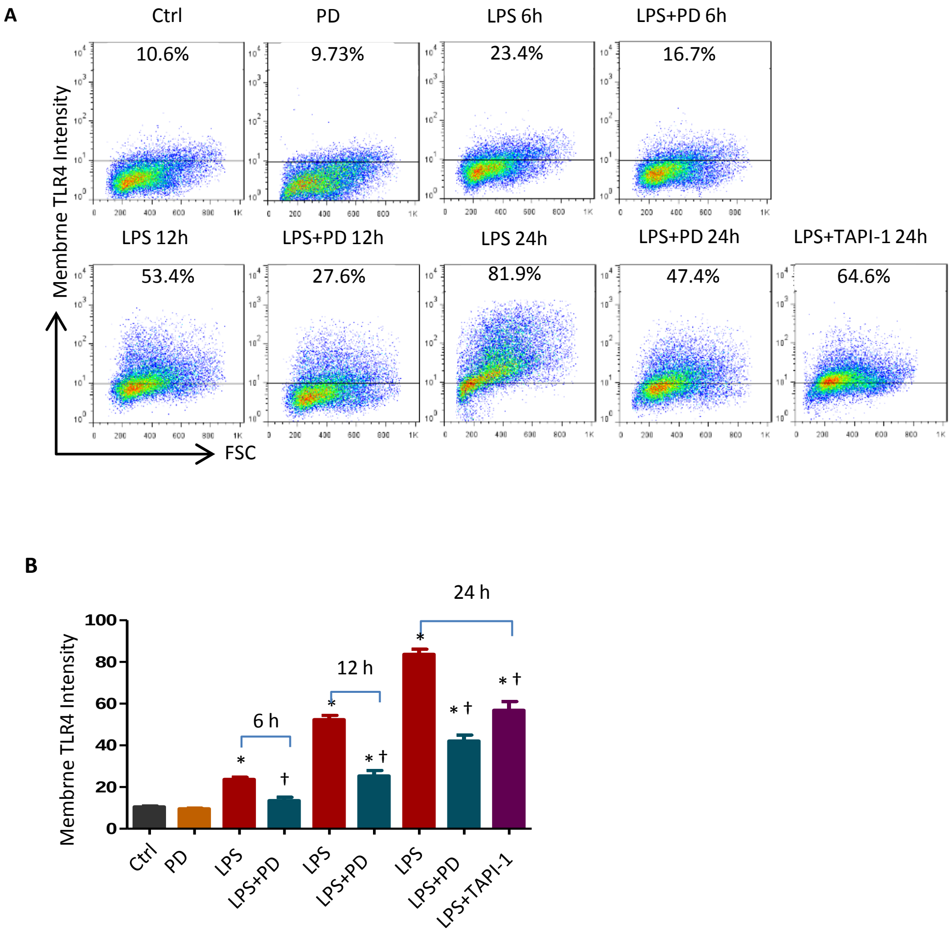

Figure S2: LPS promotes the expression of TLR4 on the cell surface of Raw264.7 cells.

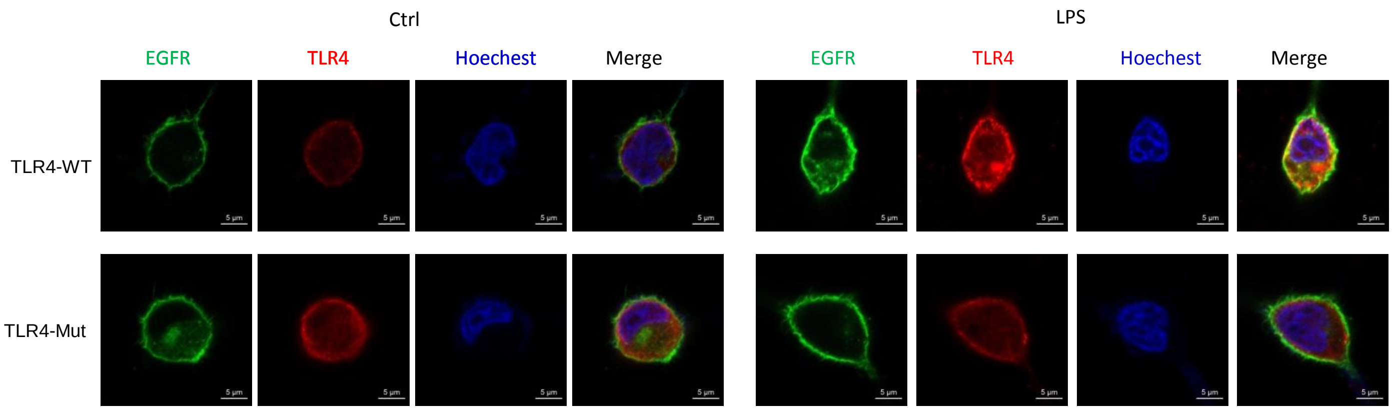

Figure S3: The effect of TLR4 phosphorylation on the colocalization between TLR4 and EGFR in response to LPS

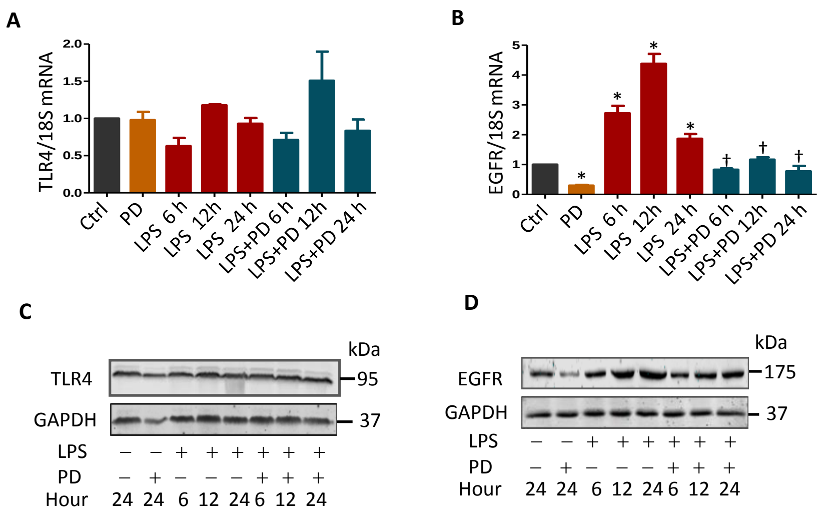

Figure S4: The effect of LPS on the expression of TLR4 and EGFR in BMDM cells.

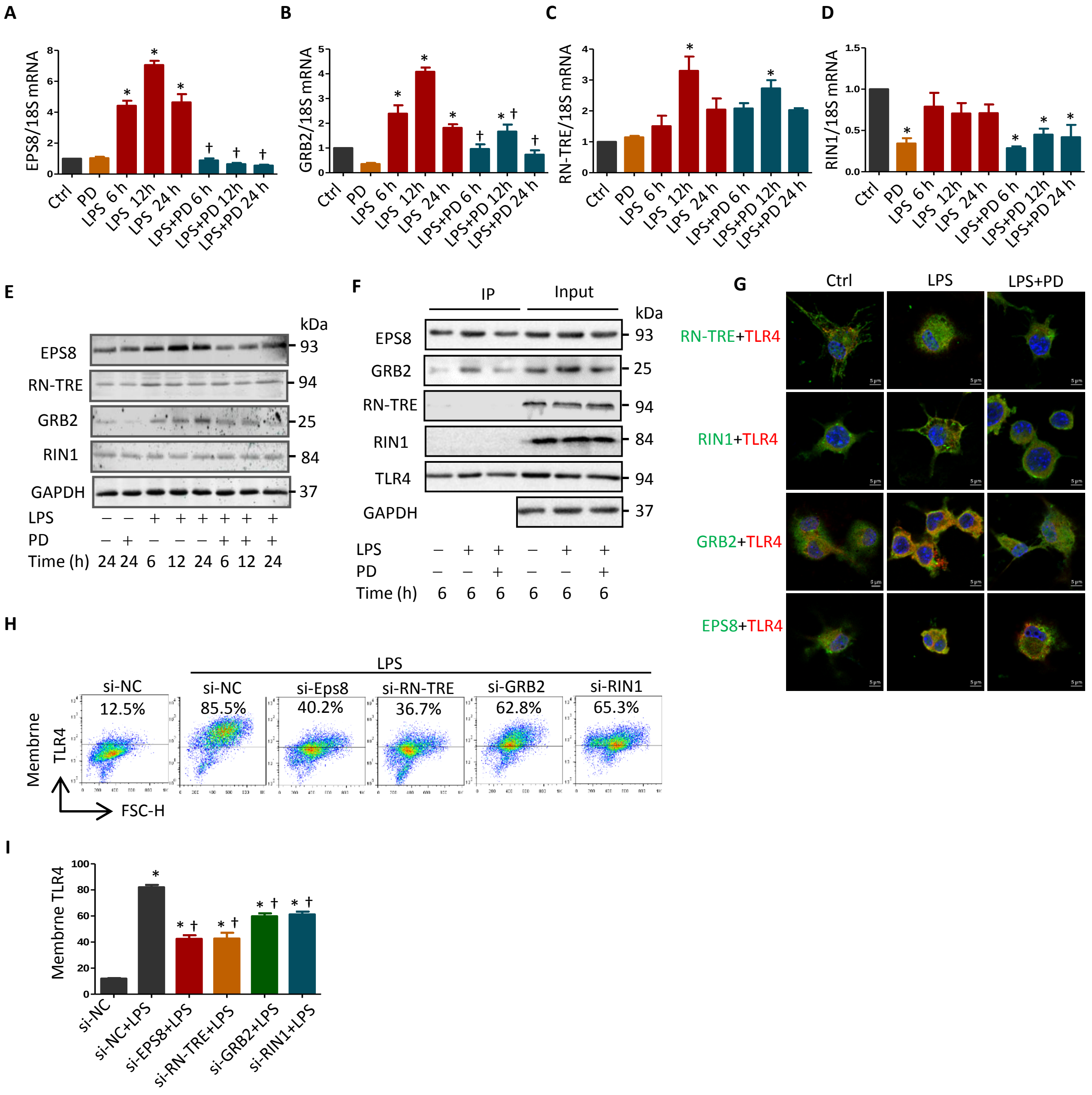

Figure S5. EPS8/GRB2/RN-TRE/RIN1 are required for the upregulation of cell surface expression of TLR4 and EGFR in response to LPS.

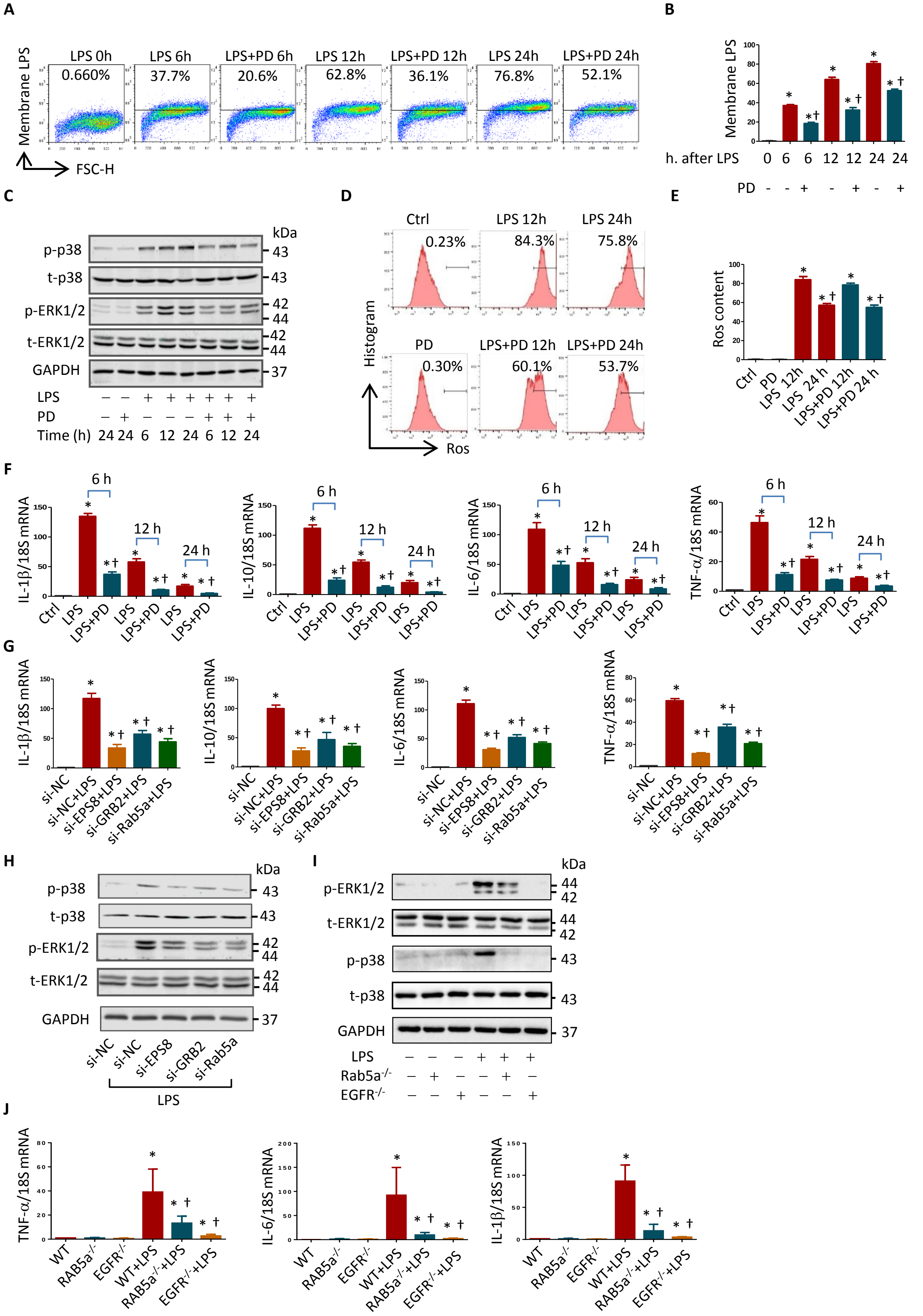

Figure S6. Upregulated cell surface TLR4 expression sensitizes cell response to LPS

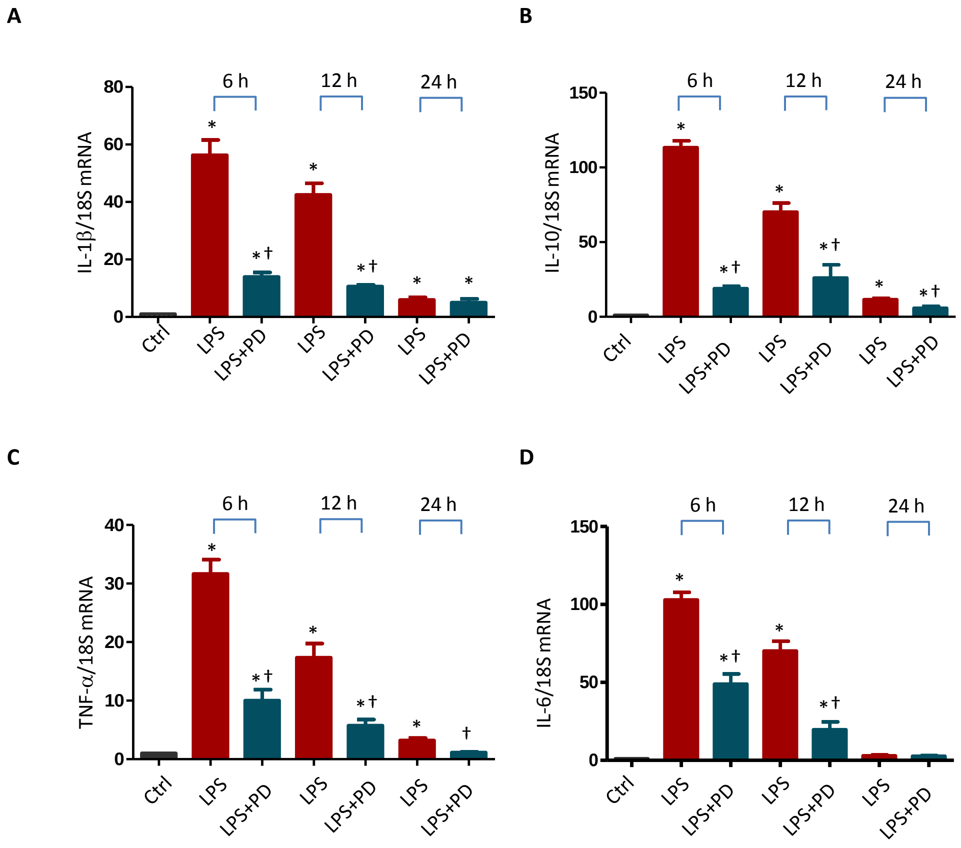

Figure S7: Increased TLR4 on the cell surface is responsible for the regulatory effect of EGFR on LPS induced cytokines production in Raw 264.7 cells.

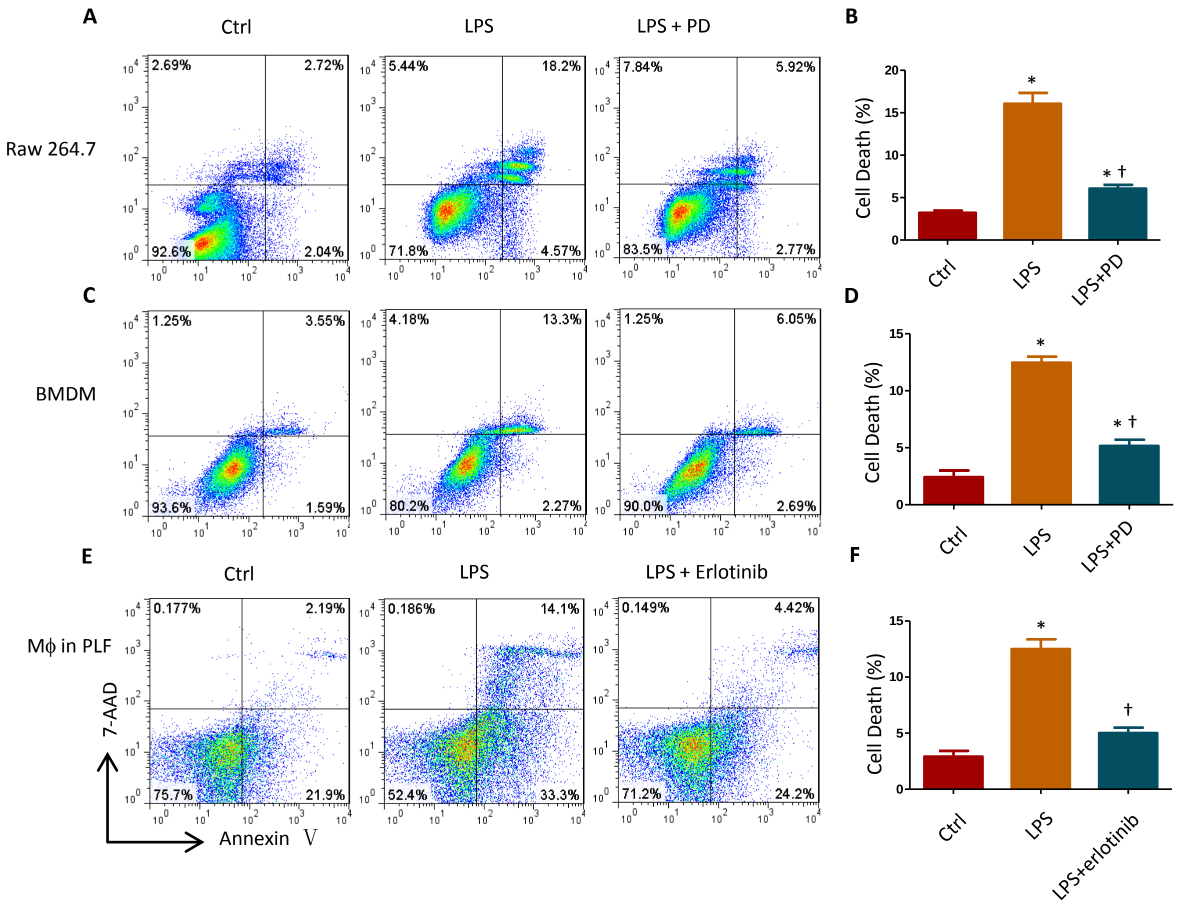

Figure S8. Upregulated TLR4 expression promotes macrophage death in response to LPS.

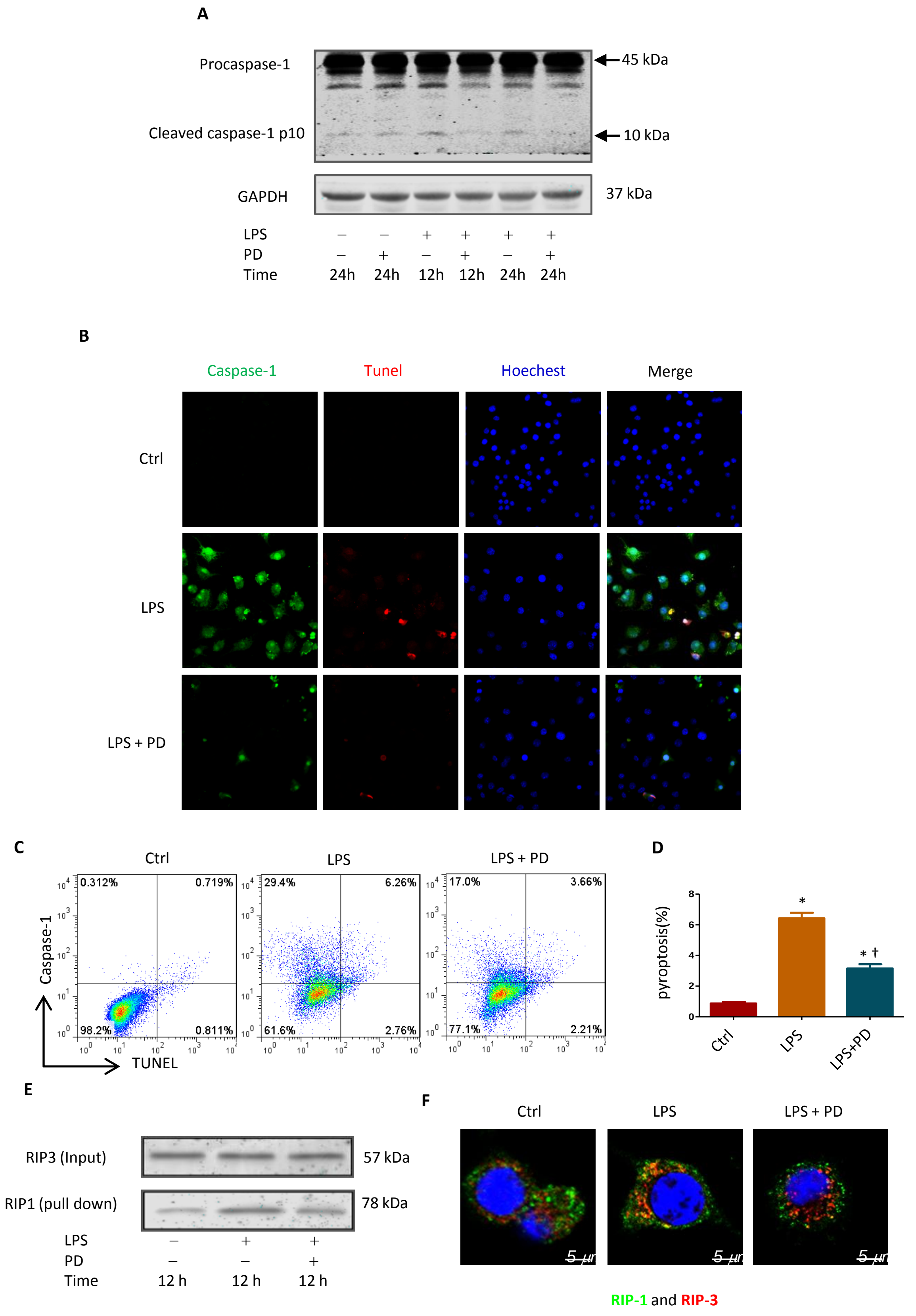

Figure S9. Upregulated TLR4 expression promotes macrophage necrosis in macrophages in response to LPS
